# Unmasking Coincident Hodgkin Lymphoma and Giant Cell Tumor: Insights from [^18^F] FDG PET/CT

**DOI:** 10.22038/AOJNMB.2023.74639.1519

**Published:** 2024

**Authors:** Akram Al-Ibraheem, Serin Moghrabi, Ahmed Saad Abdlkadir, Mohamad Haidar, Omar Jaber

**Affiliations:** 1 Department of Nuclear Medicine, King Hussein Cancer Center (KHCC), Amman, Jordan; 2 School of Medicine, The University of Jordan, Amman, Jordan; 3 Department of Diagnostic Radiology, American University of Beirut Medical Center, Beirut, Lebanon; 4 Department of pathology, King Hussein Cancer Center (KHCC), Amman, Jordan

**Keywords:** PET/CT, Hodgkin Lymphoma, Giant Cell Tumor, Synchronous Giant Cell Tumor

## Abstract

Tenosynovial giant cell tumors represent a group of typically non-malignant tumors found within the joints and soft tissues. The occurrence of tenosynovial giant cell tumor alongside hematologic malignancies is an infrequent finding. Herein, we report a patient who presented with coinciding Hodgkin Lymphoma (HL) and tenosynovial giant cell tumor before chemotherapy initiation. The case was discovered during initial assessment using [^18^F]fluorodeoxyglucose ([^18^F]FDG) positron emission tomography/computed tomography (PET/CT) imaging for HL staging. An unrelated hypermetabolic mass within the left knee joint led to the discovery of this unusual case, which led to a CT-guided biopsy and tenosynovial giant cell tumor discovery. This was clearly demonstrated in interim and end-of-therapy PET/CT studies when all lymphomatous lesions had resolved but the tenosynovial giant cell tumor remained. This case serves as a reminder of the intricate nature of oncological pathology and emphasizes the need for thorough and vigilant diagnostic evaluation for optimal management plan.

## Introduction

 The prevalence of giant cell tumors in hematologic malignancies is increasing (1). 

 Numerous instances have been documented wherein patients previously diagnosed with cancer have undergone multiple chemotherapy regimens (2). Furthermore, there have been reports of recurrent and metachronous giant cell tumors occurring in individuals with a history of heavy smoking (2, 3). Based on previous empirical findings, it can be inferred that giant cell tumors exhibit a predisposition that is influenced by multiple factors. The incidental discovery of a giant cell tumor is a common occurrence, and the absence of distinct clinical manifestations often leads to a delay in reaching a definitive diagnosis (1). This case report presents the initial documentation of a patient exhibiting synchronous giant cell tumor and Hodgkin lymphoma (HL), which was detected through whole-body [^18^F]fluorodeoxy-glucose ([^18^F]FDG) positron emission tomography/computed tomography (PET/CT) imaging. 

 Subsequently, further evaluation with histopathologic approval help unveiled the hidden harmony. Upon resuming follow-up, it became clearly evident in interim and end-of-therapy PET/CT studies when all lympho-matous lesions had resolved but the giant cell tumor remained. Therefore, nuclear medicine physicians need to be aware of this possibility when assessing irrelevant intraarticular lesion regardless of its clinical significance.

## Case Report

**Figure 1. (a -f):**
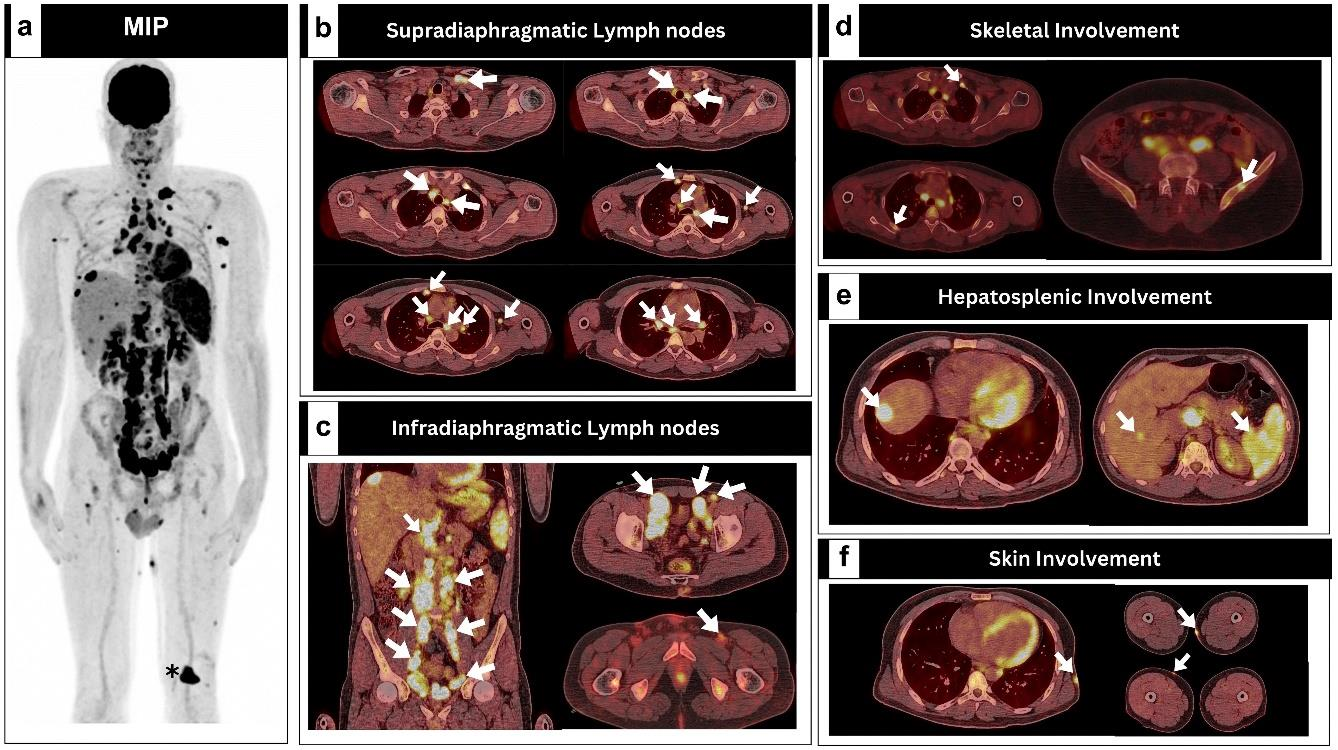
Baseline [^18^F] FDG PET/CT images demonstrating the lymphomatous disease extent (**arrows**) in conjunction to irrelevant single intraarticular 4 cm hypermetabolic mass lesion occupying the anteromedial aspect of the left knee (**asterisk**)

**Figure 2:**
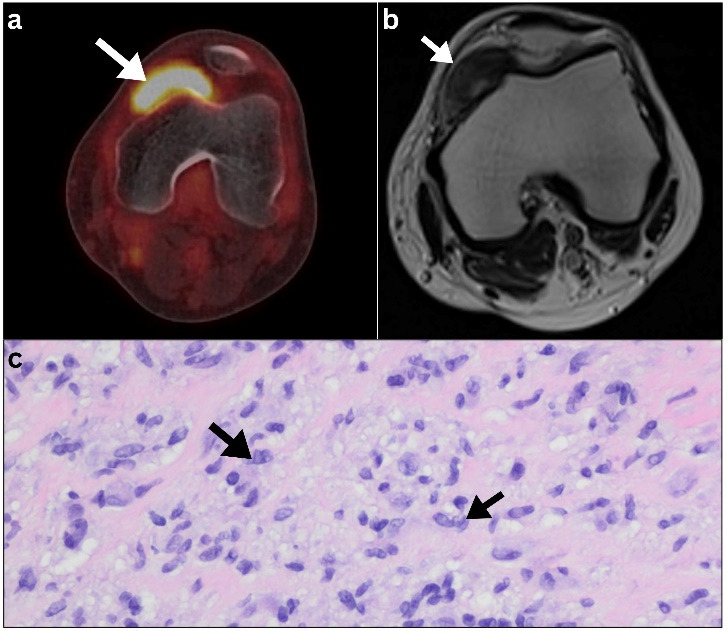
(**a**) Initial axial PET/CT image of the left knee revealed hypermetabolic mass occupying the anteromedial aspect of the left knee (**arrow**). (**b**) Initial T1 axial MRI of left knee revealed a well-defined intra-articular hypointense soft tissue mass visualized at the medial aspect of the left knee joint space with medial extension lying dorsal to the inferior pole of the patella (**arrow**). (**c**) Histopathologic examination of the synovial mass confirmed the presence of giant cell tumor without lymphomatous infiltration

 A 30-year-old heavy smoker man presented with a three-month history of fever, weight loss, and night sweats. The physical examination revealed right inguinal swelling, fever, and pallor but was otherwise unremarkable. Initial laboratory tests were all normal apart from mild anemia (hemoglobin of 10.5 g/dl). The diagnosis of HL was confirmed based on the histopathological examination of an excised inguinal lymph node, which revealed the presence of Reed-Sternberg cells. Shortly thereafter, initial [^18^F]FDG PET/CT imaging revealed hypermetabolic lymphomatous disease involving multiple groups of lymph nodes above and below the diaphragm, along with bone, skin, liver, and splenic involvement (Figure 1). Interestingly, a suspicious yet irrelevant single intraarticular 4 cm hypermetabolic mass was observed in the anteromedial aspect of the left knee (Figure 2a). The maximum standardized uptake value (SUV_max_) was 14.1. This lesion was deemed suspicious with radiologic and metabolic features that seem unattributable to the potential lymphomatous disease, mandating further evaluation. Therefore, orthopedic examination of left knee was done and revealed mild swelling in the area with no attributable pain, tenderness, or effusion and with normal range of motion. Subsequently, MRI of the left knee (Figure 2b) showed a well-defined mass with specific imaging characteristics consistent with a tenosynovial giant cell tumor. A biopsy of the synovial mass was performed to rule out lymphomatous infiltration, confirming the presence of a tenosynovial giant cell tumor without lymphomatous infiltration (Figure 2c). 

**Figure 3:**
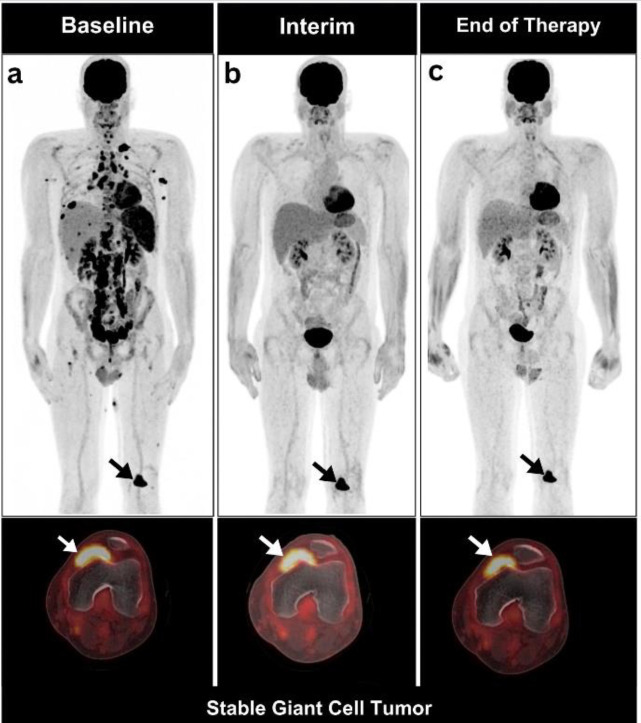
Serial Maximum Intensity Projection images, with their corresponding fused axial PET/CT of the knee (**a**) performed before chemotherapy, (**b**) following 3 cycles of chemotherapy (interim), and (**c**) following the end of therapy demonstrating picture of complete response in Hodgkin Lymphoma but with stable giant cell tumor (**arrows**)

 Afterwards, the patient's case was discussed in a multidisciplinary clinic, and a decision was reached to postpone the surgical removal of the giant cell tumor until the completion of chemotherapy cycles for HL. A total of six cycles of Adriamycin, Bleomycin, Vinblastine, and Dacarbazine (ABVD) chemotherapy were received. Following the third cycle (interim period) and beyond, the patient achieved complete HL remission with an otherwise stable giant cell tumor, as depicted by follow-up [^18^F]FDG PET/CT (Figure 3). His laboratory tests were also unremarkably normal from this timeframe and beyond. During that time, the patient was convinced to quit smoking after consultation of smoking cessation clinic. The patient is presently in the process of scheduling a wide local excision procedure for the giant cell tumor and will subsequently undergo regular monitoring and evaluation.

## Discussion

 When assessing hypermetabolic lesions within a joint, it is essential to thoroughly analyze the pattern of uptake and associated risk factors for the disease. Intraarticular lymphomatous deposits typically occur near sites of epiphyseal metastases, leading to concurrent inflammation or structural damage (4). These deposits often have unclear boundaries and show a diffuse pattern of [^18^F]FDG uptake (5). In our specific case, there was no evidence of localized deposition in the adjacent bone structures, and there was no joint pain or tenderness indicating inflammation. The well-defined and distinct margins of the intraarticular mass strongly trigger a spotlight on synovial pathology rather than lympho-matous deposits as the primary consideration. 

 This was further supported later on interim and end-of-therapy PET/CT studies where all lymphomatous lesions had resolved but the giant cell tumor remained. Typically, Intra-articular synovial tumors with moderate FDG uptake are characteristic of giant cell tumor (6). Intense FDG uptake is rare but can pose a diagnostic challenge, mimicking sarcomatous or metastatic conditions (6). 

 Therefore, it is essential to perform site-specific tissue confirmation to rule out synchronous occurrences when stable non-responsive regions are observed during the interim stage and beyond (7).

 Generally, giant cell tumor is an infrequent benign bone tumor with an unclear cause (1). It predominantly affects the knee joint, while other locations such as the hips, ankle, shoulder, and elbow follow in decreasing frequency (1). 

 Giant cell tumor are commonly non-malignant and localized to a specific region (8). However, there have been rare occurrences of multicentric 

giant cell tumor with pulmonary spread, which can complicate the initial identification and treatment of the condition (8). Giant cell tumor is frequently discovered incidentally, and the lack of specific clinical manifestations often delays a conclusive diagnosis (1). In our instance, the patient did not present any symptomatic indications that would warrant further investigation at the site affected by giant cell tumor. However, [^18^F]FDG PET/CT findings paired with high clinical suspicion and multidisciplinary collaboration were helpful in reaching the diagnosis quickly and effectively.

 In giant cell tumor, the type of surgical intervention depends on the tumor demarcation and often involves wide local excision for well-defined tumors (1). When giant cell tumor coincides with HL before chemotherapy induction, it is necessary to pursue a multidisciplinary approach since chemotherapy administration and active smoking history can complicate the post-operative period (9). These problems can be mitigated by delaying surgical intervention for giant cell tumor until conclusion of chemotherapy and preferably convincing the patient to quit smoking at least six weeks before surgery establishment (9).

 The recognition of giant cell tumor in hematologic malignancies is growing. To our knowledge, this is the first documented occurrence of giant cell tumor in a patient with HL prior to starting chemotherapy. Therefore, nuclear medicine physicians should be mindful of this potential when examining distant and unrelated hypermetabolic lesions, whether for staging, evaluation, or follow-up.

## Compliance with Ethical Standards

 Funding: The authors received no financial support for the research, authorship or publication of this article.

## Ethical approval

 All procedures performed in studies involving human participants were in accordance with the ethical standards of the institutional and/or national research committee and with the 1964 Helsinki declaration and its later amendments or comparable ethical standards.

## Informed Consent

 Informed consent was obtained from the patient for publication of his case/report and accompanying images. 
